# A Whiff of Tobacco and Its Unpleasant Suggestion

**Published:** 1898-11

**Authors:** 


### A Whiff of Tobacco and Its Unpleasant Suggestion.

We do not want to rail at tobacco, but just to draw a little moral from a whiff of smoke. Go where one will, the nose, the eyes, and even the throat and bronchial tubes, are assailed by smoke, that is, by a cloud of particles, every one of which has come direct out of some one else's mouth. It is not a nice idea, but let us see what else it teaches. The fumes do but make visible what is happening all the day, whether we smoke or not. Each of the tiny particles of carbon or condensed vapor, which in their millions make up a wreath of smoke, does but indicate the track taken by a corresponding particle of expired air, which, if it can carry the visible carbon, can still more easily carry the invisible microbe. Thus a whiff of smoke entering our nostrils and penetrating our lungs does but show the course which might be taken just as easily by a swarm of microbes, and serves to demonstrate one at least of the ways in which a crowded life passed in close community with our fellows leads to mischief. The passage of a whiff of smoke from mouth to mouth does, in fact, but illustrate the mode in which the well-recognized evils of rebreathing expired air are produced. It is not the air, but what the air carries with it that does the harm. What is illustrated by tobacco smoke is sometimes proved in another way. In the bright sunbeams motes are said to dance, and by careful watching one may see not only how numerous these motes are, but of what nasty stuff they are not infrequently composed. The wheezy flower seller coughing over his tray of violets, the loud-voiced hawker shouting over his barrow of strawberries, the sniffling child sneezing at the street corner, the panting person who will shake out his handkerchief in the 'bus before using it, even polite people talking to each other, are all doing things which on a dull day seem innocuous enough. Let the sun shine, however, and the telltale sunbeams soon display the showers of saliva and the clouds of dust which are thus scattered in the air and can almost be traced from mouth to mouth. This is esthetically abominable, but in the vast majority of cases probably does no harm. Here and there, however, these particles come from people who are diseased, and carry diseases to those who are healthy. The rebreathing of expired air is certainly one cause of disease, especially to those who live in towns and in close dwellings; and how real is the risk, and how readily the passage of solid particles from man to man and from mouth to mouth is accomplished, is made manifest every time a whiff of tobacco makes us cough.—*The Hospital.*

